# Implementation of antiretroviral therapy guidelines for under-five children in Tanzania: translating recommendations into practice

**DOI:** 10.7448/IAS.18.1.20303

**Published:** 2015-12-18

**Authors:** Harriet Nuwagaba-Biribonwoha, Chunhui Wang, Bonita Kilama, Farhat K Jowhar, Gretchen Antelman, Milembe F Panya, Elaine J Abrams, for the Identifying Optimal Models of HIV Care in Africa: Tanzania and Zanzibar Consortium

**Affiliations:** 1 ICAP at Columbia University, Mailman School of Public Health, Columbia University, New York, NY, USA; 2 Department of Epidemiology, Mailman School of Public Health, Columbia University, New York, NY, USA; 3 National AIDS Control Program, Dar Es Salaam, Tanzania; 4 Zanzibar AIDS Control Program, Zanzibar, Tanzania

**Keywords:** antiretroviral therapy, HIV-infected children, infants, ART cascade, Tanzania

## Abstract

**Introduction:**

Paediatric antiretroviral therapy (ART) guidelines have been updated several times in recent years. We assessed implementation of ART guidelines among under-five children to inform the transition to universal paediatric ART in Tanzania.

**Methods:**

We conducted a retrospective cohort analysis of infants (0 to 11 months) and children (12 to 59 months) enrolled between 2010 and 2012 using routinely collected data. Infants and children were initiated on ART according to the 2008 World Health Organization (WHO) recommendations/2009 Tanzania guidelines (universal ART for infants). Cumulative ART initiation incidence and correlates of ART initiation were examined using competing risk methods accounting for attrition (death or loss to follow-up). Kaplan-Meier methods and Cox regression models were used to examine attrition on ART and its correlates.

**Results:**

A total of 1679 children were enrolled at 69 clinics: 469 (28%) infants and 1210 (74%) children. Infant cumulative ART initiation incidence was 59.6, 71.3 and 78.0% at one, three and six months of follow-up. Infants were more likely to start ART if enrolled in 2012 [adjusted sub-hazard ratio (AsHR)=2.2, 95% confidence interval (CI): 1.7 to 2.8] or 2011 (AsHR=1.8, 95% CI: 1.4 to 2.3) compared to 2010; they were more likely to start ART from prevention of mother-to-child HIV transmission (AsHR=1.6, 95% CI: 1.3 to 2.1) and inpatient wards (AsHR=1.5, 95% CI: 1.2 to 2.0) versus being enrolled from voluntary counselling and testing centres. Attrition at 12 months on ART was 33.9% and was more likely among infants with WHO Stage 4 [adjusted hazard ratio (AHR)=3.1. 95% CI: 1.8 to 5.2] and severe malnutrition (AHR=1.4, 95% CI: 1.0 to 1.9).

Among 599 children eligible for ART at enrolment, cumulative ART initiation incidence was 51.8, 68.6 and 76.1% at one, three, and six months. Children were more likely to start ART if enrolled in 2012 (AsHR=1.8, 95% CI: 1.4 to 2.3) or 2011 (AsHR=1.5, 95% CI: 1.2 to 1.8) compared to 2010; they were more likely to start ART at primary health facilities (AsHR=1.5, 95% CI: 1.1 to 2.0) and less likely at urban facilities (AsHR=0.6, 95% CI: 0.5 to 0.9) and facilities without CD4 testing on site (AsHR=0.7, 95% CI: 0.5 to 0.9). Attrition at 12 months on ART was 23.1% and was more likely with severe malnutrition (AHR=1.8, 95% CI: 1.1 to 3.0), WHO Stage 4 (AHR=3.0, 95% CI: 1.0 to 8.5) and outpatient enrolees (AHR=1.7, 95% CI: 1.1 to 2.7).

**Conclusions:**

Our findings suggest the gradual adoption of guidelines over calendar time. Interventions to expedite ART initiation and support retention on ART are needed.

## Introduction

There is global acknowledgement that children are being left behind in the antiretroviral therapy (ART) scale-up [1, 2]. In 2013, 38% of all adults living with HIV were receiving ART compared to only 24% of children under 15 years of age [3, 4]. The disparities in paediatric ART access have prompted global interventions to maximize the number of children on treatment. There are new targets to get 90% of all people living with HIV, including children, on ART [5] and specific initiatives to promote paediatric ART access and double the number of children on treatment [6, 7].

The United Republic of Tanzania (Tanzania) faces similar challenges with paediatric ART scale-up. It is estimated that 16,000 new HIV infections occurred in 2012 among children <15 years in 2013 [3]. This represents a 50% reduction in the new infections recorded five years prior, but more than 300,000 children are estimated to be living with HIV in the country [3, 8], the majority having acquired it through vertical transmission. Notably, only an estimated 16% of children are receiving ART, less than half the estimated 41% of adults on treatment in Tanzania [3].

Children need to be prioritized in the ART scale-up process because of the high risk of mortality and loss to follow-up, both before and after ART initiation [9–11]. Over the past five years, the World Health Organization (WHO) has recommended that ART be universally initiated for children in the most vulnerable age groups, regardless of clinical or immunologic disease stage. In 2008 this recommendation applied to all children under one year; it was expanded in 2010 to include all children under two years, and in 2013 it was recommended that all children under five years of age initiate ART [12–14]. In this context of rapidly changing guidance and accelerated global efforts to increase the number of children with ART, it is important to assess to what extent previous recommendations translated into changes in clinical practice and improved outcomes for children. We examine this in Tanzania, where the Ministry of Health (MoH) adopted the 2008 and 2010 WHO recommendations in their 2009 and 2012 paediatric treatment guidelines, respectively [15, 16].

## Methods

We conducted a retrospective cohort analysis of de-identified routinely collected data as part of the Identifying Optimal Models of HIV Care in Africa study. The Optimal Models study methods have been previously described [9, 17, 18]. Briefly, ICAP at Columbia University through funding from the President's Emergency Plan for AIDS Relief (PEPFAR) provided technical support to clinics in three regions of mainland Tanzania (Kagera, Kigoma, Pwani) and Zanzibar to provide a standard package of HIV care and treatment services. As patients accessed care, routinely collected data about patient demographic characteristics, clinical assessments and treatment outcomes were captured in paper records and later entered into on-site electronic databases. Data cleaning was performed and the dataset de-identified and exported for analysis.

### Health facility context

Information about clinic and programme characteristics was collected annually using a health facility assessment questionnaire. Data from the 2011 health facility assessment questionnaire were used for this analysis. Health facilities were categorized as primary (including public health centre, dispensaries and clinics) and secondary/tertiary health facilities, which included public district or regional hospitals. Private health facilities included hospitals and a few health centres mainly managed by faith-based organizations and non-governmental organizations as well as mixed private-public health facilities. All secondary/tertiary health facilities and some private facilities had in-patient wards, but primary health facilities, specifically dispensaries, generally provided outpatient care. Health facilities provided paediatric ART services integrated with adult services in a family-focused care model, but few secondary high volume health facilities had paediatric-friendly areas in the ART clinic or had paediatric-focused days. DNA PCR for early infant diagnosis was collected at some health facilities, with analysis done in one central lab in a different region (Mwanza). Some health facilities had FaCS Calibur/Count machines to conduct CD4 cell count testing on site, but at other facilities samples were collected on site and transported to another health facility for testing.

### Routine care and patient-level data collected

Children accessed HIV services through multiple entry points: voluntary counselling and testing (VCT) of parents and guardians in the outpatient section of the health facility and through community mobilization initiatives, prevention of mother-to-child HIV transmission (PMTCT) services offered in postnatal clinics, provider-initiated testing and counselling provided as part of outpatient and inpatient care and other entry points like tuberculosis clinics. HIV care and treatment for children was mainly provided by medical officers and medical assistants, with paediatricians only available at a few regional hospitals and some faith-based secondary health facilities. Nurse-led ART initiation prescription and initiation were not yet implemented in Tanzania. ART was usually dispensed by pharmacy assistants or nurses. HIV care and treatment services, including CD4 cell count testing and ART, were freely provided at public health facilities, though some private facilities charged minimal token fees, generally under $2.

At the first clinic visit (enrolment visit), demographic characteristics (age, gender, source of referral) were captured and a clinical assessment done (weight in kilograms with one decimal place, WHO clinical staging, CD4 cell count; CD4 percent was not routinely measured). Monthly follow-up visits were recommended for children followed before (pre-ART) and after ART initiation. At follow-up visits, the clinical and ART status of each child was reviewed and documented and appropriate clinical management (including ART initiation for those eligible) provided as per national guidelines. CD4 cell count testing to assess ART eligibility was to be repeated every six months, or more frequently if there was a clinical indication for it. Where a CD4 cell count machine was available at the health facility, results were generally available the next day. Health facilities without a CD4 cell count machine waited about a week for return of results, depending on the distance and availability of transport to and from the testing facility.

This analysis includes all ICAP-supported clinics in Tanzania with electronic databases that enrolled children aged 0 to 59 months between 1 January 2010 and 30 September 2012. During this time, ART was initiated according to the 2008 WHO recommendations/2009 Tanzania guidelines, which recommended ART initiation for all infants (0 to 11 months); all children with WHO Stage 3 or 4 irrespective of CD4 cell count; children aged 12 to 35 months with CD4 cell count <750 cells/µL; and children aged 36 to 59 months with CD4 cell count <350 cells/µL irrespective of the WHO stage [16]. The years of observation in this analysis were selected to evaluate implementation of these guidelines. Only ART-naïve children were included in the analysis; children who were already on ART before they were enrolled were excluded. Children's ART eligibility was evaluated at enrolment and at each follow-up visit, until they reached ART eligibility. The data set was closed on 31 March 2013, which allowed each child to have at least six months of follow-up by the time of data analysis.

### Data analysis

We categorized children <5 years as *infants* (0 to 11 months), *younger children* (12 to 35 months) and *older children* (36 to 59 months). Because visit schedules were similar for all pre-ART and ART children aged <5 years, we defined *lost to follow-up* as not dead, not transferred out and with no documented clinic visit for >180 days for all children. *Attrition* was defined as recorded death or loss to follow-up. Children who did not have the attrition outcome (i.e. who were not dead or lost to follow-up) were categorized as *retained*: these children had made a clinic visit within 0 to 180 days preceding database closure. Children who transferred out were censored at the date of transfer.

We assessed for the following outcomes: (1) Among infants 0 to 11 months of age: cumulative incidence of ART initiation and factors associated with ART initiation, as well as correlates of attrition after ART initiation; (2) among children 12 to 59 months of age: ART eligibility assessment, cumulative incidence of ART initiation and factors associated with ART initiation among those children who were ART-eligible at enrolment and correlates of attrition after ART among all older children who initiated ART. We describe progression to ART eligibility and initiation for children not ART eligible at enrolment and provide supplemental digital content on correlates of pre-ART attrition among all infants and children, as well as factors associated with being ART eligible at enrolment among children.

We conducted descriptive analyses to summarize the characteristics of HIV-infected infants and children, overall and by age group (infants, younger and older children). Chi-square tests were used to analyze the differences between age groups. Logistic regression was applied to assess the predictors of ART eligibility at enrolment among children 12 to 59 months, using generalized estimating equations (GEE) to account for within-facility correlations. Among infants and ART-eligible children, competing risk methods were used to estimate the cumulative ART initiation incidence and factors associated with ART initiation, accounting for the risks of death and loss to follow-up. Crude and adjusted sub-distribution hazard ratios (AsHRs) were calculated to assess the association between patient-level and facility-level factors and ART initiation. To evaluate attrition and the predictors of attrition among HIV-infected infants and children who started ART, Kaplan-Meier analyses were conducted and Cox-regression was used to calculate the hazard ratios (HRs). Finally, to assess pre-ART attrition among all infants and children enrolled, competing risk methods accounting for ART initiation were used. Multivariate models included age group, sex, point of entry, enrolment year, WHO stage and malnutrition (using weight-for-age *z*-scores) at enrolment, facility type, location and services (nutrition support, outreach programmes and having CD4 cell count machine on site) as potential confounding variables. Analyses were performed using SAS 9.3 (SAS Institute, Cary, NC, USA) and Stata 12.1 (Stata Statistical Software, Stata Corp, College Station, TX, USA).

### Ethical approval

The Optimal Models study was reviewed and approved by the National Institute for Medical Research of Tanzania, Zanzibar Medical Research and Ethics Committee, Columbia University Medical Center Institutional Review Board, US Centers for Disease Control and Prevention and the US Office of the Global AIDS Coordinator. There was no interaction with children during the study and individual consent/assent was waived by all approving boards.

## Results

**Figure 1:**
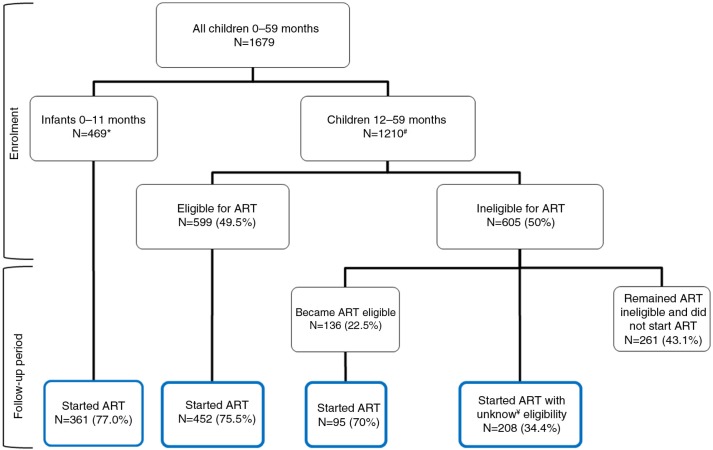
Antiretroviral therapy (ART) eligibility and ART initiation among children less than five years of age enrolled in HIV care between 2010 and 2012 in Tanzania. *All infants were ART eligible. ^#^ART eligibility assessment was mainly by WHO staging, six children had insufficient records to determine ART eligibility. ^¥^Unknown eligibility means there was no follow-up documentation of WHO stage or CD4 cell count to categorize eligibility.

Between January 2010 and September 2012, a total of 1679 children were enrolled at 69 health facilities: 469 (28%) infants (0 to 11 months), 780 (46%) younger children (12 to 35 months) and 430 (26%) older children (36 to 59 months) (Figure 1). The 1679 children contributed a total of 1880 child-years in follow-up. The overall median age was 1.7 years [interquartile range (IQR), 0.9 to 3.1], and 50.4% were male (Table 1). Most of the children were enrolled from urban clinics (78.4%). Over two-thirds were enrolled at public primary (34.8%) and secondary (36.9%) health facilities, while 28.2% were enrolled at private and other facilities. Half the children (53.4%) attended clinics with a CD4 cell count machine on site and the majority (92.9%) attended health facilities that offered early infant diagnosis by dried blood spot collection with analysis at a lab off site. A small proportion of children (17.9%) were enrolled at clinics where food rations were provided to children, and 89.1% were enrolled at clinics providing outreach services for adult and paediatric patients who miss appointments.

**Table 1:** Enrolment characteristics by age group of HIV-infected children less than five years of age enrolled in HIV care between January 2010 and September 2012 in Tanzania

	All under-five children		Infants (0 to 11 months)		Young children (12 to 35 months)		Older children (36 to 59 months)		
					
Patient-level characteristics	*n*=1679	%	*n*=469	%	*n*=780	%	*n*=430	%	*p*
Sex									
Male	846	50.4	217	46.3	408	52.3	221	51.4	0.11
Female	833	49.6	252	53.7	372	47.7	209	48.6	
Point of entry into care									
VCT	567	33.8	92	19.6	291	37.3	184	42.8	<0.0001
PMTCT	456	27.2	234	49.9	171	21.9	51	11.9	
Inpatient	147	8.8	51	10.9	71	9.1	25	5.8	
Outpatient	280	16.7	53	11.3	129	15.5	98	22.8	
Other/unknown	229	13.6	39	8.3	118	15.1	72	16.7	
Transferred in from another facility	139	8.3	7	1.5	70	9.0	62	14.4	<0.0001
Enrolment year									0.04
2010	607	36.2	156	33.3	273	35.0	178	41.4	
2011	559	33.3	154	32.8	263	33.7	142	33.0	
2012	513	30.6	159	33.9	244	31.3	110	25.6	
Malnutrition (weight for age)									<0.0001
Missing	50	2.9	17	3.3	20	2.6	13	3.0	
Not malnourished^a^	754	46.3	268	59.3	260	34.2	226	54.2	
Moderately malnourished^a^	305	18.7	69	15.3	169	22.2	67	16.1	
Severely malnourished^a^	570	35.0	115	25.4	331	43.6	124	29.7	
WHO stage									<0.0001
Missing	29	1.7	15	3.2	9	1.2	5	1.2	
Stage 1	474	28.7	168	37.0	183	23.7	123	28.9	
Stage 2	489	29.6	113	24.9	231	30.0	145	34.1	
Stage 3	442	26.8	107	23.6	230	29.8	105	24.7	
Stage 4	245	14.9	66	14.5	127	16.5	52	12.2	
CD4 count (cells/µL)									0.02
Missing	1316	78.4	407	86.8	621	79.6	288	67.0	
1 to 350	99	27.3	27	43.6	39	24.5	33	23.2	
351 to 750	89	24.5	13	21.0	35	22.0	41	28.9	
750+	175	48.2	22	35.5	85	53.5	68	47.9	
ART eligibility at enrolment									<0.001
Eligible	1068	63.8	469	100.0	415	53.5	184	42.9	
Not eligible	605	36.2	–	–	360	46.5	245	57.1	
**Distribution of children by facility-level characteristics (number of facilities=69)**									
Setting									0.095
Urban/semi-urban city (*n*=48)	1316	78.4	362	77.2	601	77.1	353	82.1	
Rural (*n*=21)	363	21.6	107	22.8	179	23.0	77	17.9	
Facility type									0.0002
Public primary (*n*=28)	585	34.8	165	35.2	272	34.9	148	34.4	
Public secondary/tertiary (*n*=17)	620	36.9	152	32.4	276	35.4	192	44.7	
Others, including 18 private clinics (*n*=24)	474	28.2	152	32.4	232	29.7	90	20.9	
CD4 machine at facility									0.14
Available on site (*n*=22)	897	53.4	236	50.3	416	53.3	245	57.0	
Not available on site (*n*=47)^b^	782	46.6	233	49.7	364	46.7	185	43.0	
Early infant diagnosis sample collection^c^									0.008
On site (*n*=63)	1559	92.9	421	89.8	736	94.4	402	93.5	
Not available (*n*=6)	120	7.2	48	10.2	44	5.6	28	6.5	
Nutritional support									0.03
No food rations for children (*n*=58)	1379	82.1	398	84.9	620	79.5	361	83.9	
Food rations provided to children (*n*=11)	300	17.9	71	15.1	160	20.5	69	16.1	

a Weight-for-age *z*-score: not malnourished (*z*-score≥−2); moderately malnourished (*z*-score =−2 to −3); severely malnourished (*z*-score≤−3);

b sample collected on site but analysis done at a lab off site;

c DNA PCR sample collection. All DNA PCR analysis was conducted at a central laboratory in a different region (Mwanza). VCT, voluntary counselling and testing; PMTCT, prevention of mother-to-child HIV transmission; ART, antiretroviral therapy.

### Characteristics of infants

Half (49.9%) of the 469 infants were enrolled from PMTCT clinics (Table 1). Median infant age at enrolment was seven months (IQR 4.8 to 9.4). The proportion of infants enrolled was evenly distributed by calendar year. At enrolment 38.1% had WHO Stage 3 or 4 disease. The majority (86.8%) did not have CD4 cell count data, and of the 62 that did 64.6% had a CD4 cell count <750 cells/µL. Based on weight-for-age *z*-scores, 25.4% of infants were severely malnourished while 15.3% were moderately malnourished and 59.3% had no malnutrition. Seven infants (1.5%) transferred into study clinics from other health facilities.

### Characteristics of children

Of 780 younger children and 430 older children, the majority were enrolled from VCT clinics: 37.3% of younger children and 42.8% of older children (Table 1). Among younger children 35.0% were enrolled in 2010, 33.7% in 2011 and 31.3% in 2012; the respective percentages in older children were 41.4, 33.0 and 25.5%. A higher proportion of younger children had WHO Stage 3/4 disease: 46.3% compared to 36.9% of older children (*p*<0.001). A higher proportion of young children were severely malnourished: 43.6% compared to 29.7% of older children. Nine percent of younger children transferred into study clinics, compared to 14.4% of older children.

### ART eligibility assessment

All infants were ART eligible as per prevailing guidelines. Nearly all children had WHO staging done at enrolment (Table 1). However, only 159 (20.4%) younger children and 142 (33.0%) older children had a CD4 cell count at enrolment: 46.5% of younger children had a CD4 cell count less than 750 cells/µL, while 23.2% of older children had a CD4 cell count less than 350 cells/µL. Combining WHO stage and CD4 cell count data, ART eligibility at enrolment was assessed for all but six children (99.5%): over half the younger children (53.6%) and 42.9% of older children met ART eligibility criteria for enrolment. In multivariable analyses controlling for patient and facility level factors, younger children were more likely to be ART eligible than older children [adjusted odds ratio (AOR)=1.5, 95% confidence interval (CI): 1.2 to 2.0] (supplemental digital content, Supplementary Table 1). Other factors associated with increased odds of being ART eligible were being an inpatient at enrolment, compared to those enrolled from VCT (AOR=2.3, 95% CI: 1.6 to 3.3), and attending urban clinics (AOR=2.1, 95% CI=1.3 to 3.5).

### ART initiation among infants and children

**Figure 2:**
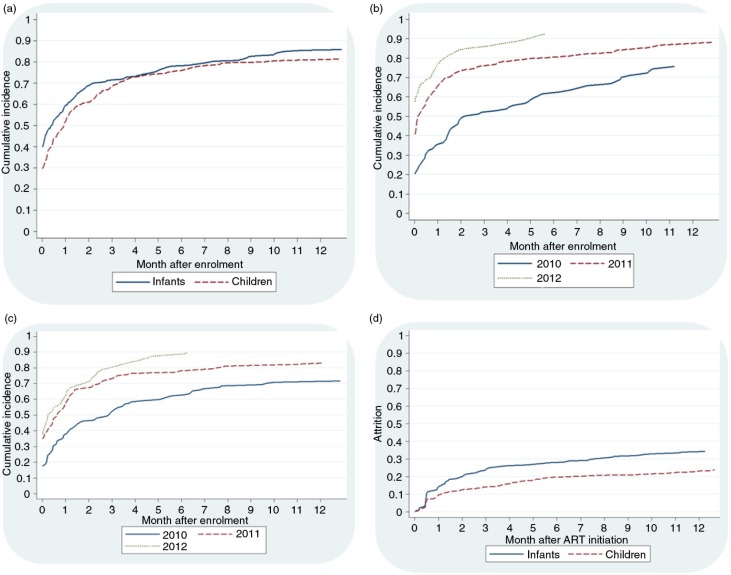
Antiretroviral therapy (ART) initiation and retention among infants 0 to 11 months and children 12 to 59 months enrolled between 2010 and 2012 in Tanzania. (a) Cumulative ART initiation among infants and ART-eligible children 12 to 59 months in Tanzania, *p*=0.15; (b) cumulative ART initiation among infants by calendar year of enrolment, *p<*0.0001; (c) cumulative ART initiation among ART-eligible children 12 to 59 months by calendar year of enrolment, *p<*0.0001; (d) attrition among infants and children 12 to 59 months who initiated ART during the study, *p<*0.0001.

Of the 469 infants, 361 (77.0%) started ART over the follow-up period (Figure 1). Cumulative incidence of ART initiation at 1, 3, 6 and 12 months of follow-up was 59.6, 71.3, 78.0 and 85.4%, respectively. Comparatively, among the 599 children eligible for ART at enrolment, 452 (75.5%) initiated ART during the follow-up period, and the cumulative incidence of ART initiation at 1, 3, 6 and 12 months of follow-up was 51.8, 68.6, 76.1 and 81.1%, respectively. Figure 2a compares the incidence of ART initiation among infants and children over the follow-up period and demonstrates no statistically significant differences between the two categories (*p*=0.15).

Among infants and children, the cumulative incidence of ART initiation increased by calendar year of enrolment (Figure 2b and c). In multivariable models adjusting for patient and facility-level characteristics, infants were more likely to initiate ART if enrolled in 2011 (AsHR=1.8, 95% CI: 1.4 to 2.3) and 2012 (AsHR=2.2, 95% CI: 1.7 to 2.8), in comparison to infants enrolled in 2010 (Table 2). Similarly, children were more likely to initiate ART if enrolled in 2011 (AsHR=1.5, 95% CI: 1.2 to 1.8) or 2012 (AsHR=1.8, 95% CI: 1.4 to 2.3). Compared to infants enrolled from VCT clinics, infants enrolled from PMTCT clinics were more likely to initiate ART (AsHR=1.6, 95% CI: 1.3 to 2.1) as were infants enrolled from inpatient wards (AsHR=1.5, 95% CI: 1.2 to 2.0). Infants were also more likely to initiate ART if they attended private health facilities compared to secondary health facilities (AsHR=1.6, 95% CI: 1.1 to 2.3), with a similar trend observed at primary health facilities (AsHR=1.4, 95% CI: 1.0 to 2.0). Children were less likely to initiate ART if severely malnourished (AsHR=0.7, 95% CI: 0.6 to 0.9). Compared to children attending secondary health facilities, children attending primary facilities were more likely to initiate ART (AsHR=1.5, 95% CI: 1.1 to 2.0). Children were also less likely to initiate ART if they attended a clinic without CD4 cell count testing on site (AsHR=0.7, 95% CI: 0.5 to 0.9) and if they attended urban clinics, in comparison to rural clinics (AsHR=0.6, 95% CI: 0.4 to 0.8) (Table 2).

**Table 2:** Factors associated with ART initiation among infants 0 to 11 months and children 12 to 59 months eligible for ART at enrolment in Tanzania

	Infants 0 to 11 months (*N*=469)		Children 12 to 59 months (*N*=599)	
		
	Crude sHR (95% CI)	AsHR (95% CI)	Crude sHR (95% CI)	AsHR (95% CI)
Age category				
12 to 35 months	–	–	1.1 (0.9 to 1.3)	1.1 (1.0 to 1.4)
36 to 59 months			Reference	Reference
Sex				
Male	Reference	Reference	Reference	Reference
Female	0.9 (0.8 to 1.1)	0.9 (0.7 to 1.0)	1.0 (0.9 to 1.3)	1.0 (0.9 to 1.2)
Malnutrition				
Not malnourished	Reference	Reference	Reference	Reference
Moderately malnourished	1.2 (0.9 to 1.6)	1.1 (0.9 to 1.4)	0.9 (0.7 to 1.2)	0.9 (0.7 to 1.2)
Severely malnourished	1.2 (0.9 to 1.4)	0.9 (0.7 to 1.2)	0.8 (0.7 to 1.0)	**0.7 (0.6 to 0.9)**
Enrolment year				
2010	Reference	Reference	Reference	Reference
2011	1.7 (1.3 to 2.2)	**1.8 (1.4 to 2.3)**	1.6 (1.2 to 2.0)	**1.5 (1.2 to 1.8)**
2012	2.3 (1.8 to 2.9)	**2.2 (1.7 to 2.8)**	1.8 (1.4 to 2.4)	**1.8 (1.4 to 2.3)**
Missing CD4 testing at enrolment	1.1 (0.8 to 1.5)	1.1 (0.9 to 1.5)	1.2 (0.9 to 1.6)	0.9 (0.8 to 1.1)
WHO stage				
Stage 1	Reference	Reference	Reference	Reference
Stage 2	1.0 (0.8 to 1.2)	1.1 (0.9 to 1.3)	1.4 (0.9 to 2.3)	1.2 (0.8 to 1.9)
Stage 3	1.1 (0.9 to 1.5)	1.2 (0.9 to 1.6)	1.2 (0.8 to 1.8)	1.2 (0.9 to 1.7)
Stage 4	1.0 (0.8 to 1.4)	1.2 (0.9 to 1.8)	1.1 (0.7 to 1.8)	1.2 (0.8 to 1.9)
Point of entry				
VCT	Reference	Reference	Reference	Reference
PMTCT	1.7 (1.3 to 2.1)	**1.6 (1.3 to 2.1)**	1.1 (0.7 to 1.6)	1.1 (0.8 to 1.4)
Inpatient	1.5 (1.1 to 2.0)	**1.5 (1.2 to 2.0)**	0.7 (0.5 to 1.0)	0.8 (0.6 to 1.2)
Outpatient	1.5 (1.1 to 2.0)	1.3 (0.9 to 1.8)	1.0 (0.8 to 1.4)	1.0 (0.8 to 1.4)
Other/unknown	1.6 (1.1 to 2.4)	1.5 (0.9 to 2.3)	1.3 (0.9 to 1.8)	1.2 (0.9 to 1.6)
Location				
Urban/semi-urban	0.9 (0.7 to 1.2)	1.1 (0.7 to 1.5)	0.8 (0.6 to 1.0)	**0.6 (0.4 to 0.8)**
Rural	Reference	Reference	Reference	Reference
Facility type				
Primary	1.2 (0.9 to 1.5)	1.4 (1.0 to 2.0)	1.2 (0.9 to 1.5)	**1.5 (1.1 to 2.0)**
Secondary/tertiary	Reference	Reference	Reference	Reference
Private and others	1.5 (1.1 to 2.0)	**1.6 (1.1 to 2.3)**	1.0 (0.8 to 1.3)	1.1 (0.9 to 1.3)
CD4 machine at facility				
Available on site	Reference	Reference	Reference	Reference
Not available on site	1.0 (0.8 to 1.3)	0.8 (0.6 to 1.1)	1.0 (0.8 to 1.3)	**0.7 (0.5 to 0.9)**
Nutritional support^a^	0.9 (0.6 to 1.2)	0.9 (0.6 to 1.2)	0.9 (0.6 to 1.2)	1.1 (0.8 to 1.4)

a Food rations to children. ART, antiretroviral therapy; sHR, sub-hazard ratio; AsHR, adjusted sub-hazard ratio; CI, confidence interval; VCT, voluntary counselling and testing; PMTCT, prevention of mother-to-child HIV transmission. Bold values were statistically significant.

### Attrition after ART initiation among infants and children

In survival analyses of the 361 infants who started ART, attrition was observed among 33.9% (95% CI: 29.0 to 39.3%) at 12 months after ART initiation: 18.4% (95% CI: 14.3 to 23.4%) died and 18.4% (95% CI: 14.9 to 23.8%) were lost to follow-up. A total of 755 children who started ART over the follow-up period were included in the survival analysis (including 452 eligible for ART at enrolment, 95 who became eligible over the follow-up period and 208 who started ART with insufficient follow-up data to analyze ART eligibility; Figure 1). Attrition among children at 12 months after ART initiation was 23.1% (95% CI: 20.1 to 26.5%), with death at 11.0% (95% CI: 8.8 to 13.6%) and loss to follow-up at 13.6% (95% CI: 11.1 to 16.6%). As shown in Figure 2d there was a statistically significant difference in the observed attrition between infants and children (*p*<0.0001).

In multivariable analyses, attrition was more likely if infants were severely malnourished at enrolment [adjusted hazard ratio (AHR)=1.4, 95% CI: 1.0 to 1.9] or had WHO Stage 4 disease (AHR=3.1, 95% CI: 1.8 to 5.2) (Table 3). Children were more likely to experience attrition if enrolled with WHO Stage 4 disease (AHR=3.0, 95% CI: 1.0 to 8.5), compared to those enrolled with WHO Stage 1 disease (Table 3). Attrition was also more likely among younger children compared to older children (AHR=1.6, 95% CI: 1.0 to 2.4), and among children enrolled from outpatient clinics (AHR=1.7, 95% CI: 1.1 to 2.7).

**Table 3:** Factors associated with attrition on ART among infants 0 to 11 months and children 12 to 59 months who initiated ART in Tanzania

	Infants 0 to 11 months (*n*=361)		Children 12 to 59 months (*n*=755)	
		
	Crude HR (95% CI)	Adjusted HR (95% CI)	Crude HR (95% CI)	Adjusted HR (95% CI)
Age category				
12 to 35 months			**1.6 (1.1 to 2.3)**	**1.6 (1.0 to 2.4)**
36 to 59 months			Reference	Reference
Sex				
Male	Reference	Reference	Reference	Reference
Female	0.8 (0.6 to 1.0)	0.9 (0.7 to 1.2)	0.8 (0.6 to 1.1)	0.8 (0.6 to 1.1)
Malnutrition				
Not malnourished	Reference	Reference	Reference	Reference
Moderately malnourished	1.1 (0.8 to 1.7)	0.9 (0.6 to 1.4)	1.3 (0.8 to 2.0)	1.0 (0.7 to 1.7)
Severely malnourished	**1.7 (1.3 to 2.4)**	**1.4 (1.0 to 1.9)**	**2.4 (1.5 to 3.7)**	**1.8 (1.1 to 3.0)**
ART initiation year				
2010	Reference	Reference	Reference	Reference
2011	1.3 (0.7 to 2.4)	1.5 (0.7 to 3.2)	1.2 (0.8 to 1.8)	1.1 (0.8 to 1.7)
2012	1.1 (0.6 to 2.0)	1.5 (0.8 to 2.7)	0.9 (0.7 to 1.3)	0.9 (0.6 to 1.3)
2013	1.6 (0.1 to 20.2)	2.7 (0.2 to 40.2)		
WHO stage				
Stage 1	Reference	Reference	Reference	Reference
Stage 2	**1.6 (1.0 to 2.7)**	1.6 (0.9 to 2.9)	1.2 (0.5 to 2.8)	1.3 (0.5 to 2.9)
Stage 3	**1.8 (1.1 to 3.0)**	1.6 (0.9 to 2.9)	2.0 (0.9 to 4.7)	2.2 (0.9 to 5.3)
Stage 4	**3.7 (2.2 to 6.2)**	**3.1 (1.8 to 5.2)**	**3.0 (1.1 to 8.2)**	**3.0 (1.0 to 8.5)**
Point of entry				
VCT	Reference	Reference	Reference	Reference
PMTCT	0.9 (0.5 to 1.4)	1.0 (0.6 to 2.0)	1.0 (0.6 to 1.5)	1.0 (0.6 to 1.7)
Inpatient	2.0 (0.8 to 4.9)	1.9 (0.7 to 5.2)	1.4 (0.7 to 2.7)	1.1 (0.6 to 2.2)
Outpatient	1.6 (0.8 to 3.1)	1.5 (0.8 to 2.9)	**1.6 (1.1 to 2.5)**	**1.7 (1.1 to 2.7)**
Other/unknown	0.9 (0.5 to 1.7)	1.1 (0.6 to 2.0)	0.9 (0.5 to 1.4)	0.9 (0.5 to 1.6)
Location				
Urban/semi-urban	1.1 (0.7 to 1.8)	0.7 (0.3 to 1.4)	1.2 (0.8 to 1.6)	1.1 (0.7 to 1.7)
Rural	Reference	Reference	Reference	Reference
Facility type				
Primary	0.7 (0.5 to 1.2)	0.9 (0.5 to 1.7)	1.1 (0.8 to 1.6)	1.2 (0.7 to 1.9)
Secondary/tertiary	Reference	Reference	Reference	Reference
Private and others	0.8 (0.5 to 1.3)	0.6 (0.3 to 1.3)	0.8 (0.5 to 1.2)	0.7 (0.4 to 1.1)
CD4 machine at facility				
Available on site	Reference	Reference	Reference	Reference
Not available on site	0.7 (0.5 to 1.0)	0.8 (0.4 to 1.7)	1.1 (0.8 to 1.5)	1.1 (0.7 to 1.7)
Nutritional support	1.2 (0.8 to 1.8)	0.8 (0.4 to 1.7)	1.1 (0.8 to 1.5)	1.3 (0.8 to 2.1)
Outreach services	0.8 (0.4 to 1.6)	0.8 (0.4 to 1.5)	0.8 (0.5 to 1.3)	1.0 (0.6 to 1.5)

ART, antiretroviral therapy; HR, hazard ratio; CI, confidence interval; VCT, voluntary counselling and testing; PMTCT, prevention of mother-to-child HIV transmission. Bold values were statistically significant.

### Pre-ART attrition among infants and children

We assessed pre-ART attrition among all 469 infants and 1210 children 12 to 59 months in the pre-ART phase. Among infants, pre-ART attrition at 12 months of follow-up was 18.0% (95% CI: 14.7 to 21.7%), with death in 6.5% (95% CI: 4.2 to 9.4%) and loss to follow-up in 13.6% (95% CI: 10.6 to 17.0%). Among children, pre-ART attrition at 12 months of follow-up was similar: 18.8% (95% CI: 16.6 to 21.1%) with death in 4.5% (95% CI: 3.3 to 5.98%) and loss to follow-up in 15.2% (95% CI: 13.2 to 17.4%). Multivariable analysis of factors associated with pre-ART attrition found lower risk of attrition among infants enrolled in 2012 compared to 2010 (AsHR=0.4, 95% CI: 0.2 to 0.8) and infants enrolled at private clinics compared to secondary health facilities (AsHR=0.4, 95% CI: 0.2 to 0.8) (supplementary digital content, Supplementary Table 2). The risk of pre-ART attrition among children was also lower in 2012 (AsHR=0.4, 95% CI: 0.3 to 0.6) but higher among those missing a CD4 cell count (AsHR=2.0, 95% CI: 1.4 to 2.7), those with severe malnutrition (AsHR=1.4, 95% CI: 1.0 to 1.9) and children enrolled from outpatient clinics (AsHR=1.6, 95% CI: 1.1 to 2.2) (Supplementary Table 2).

## Discussion

We examined ART eligibility assessments in children and ART initiation and retention among infants and children enrolled in HIV care and treatment programmes in Tanzania in 2010 to 2012. We found universal assessment of ART eligibility among children 12 to 59 months using WHO clinical staging, but CD4 cell count testing was limited. Cumulative ART initiation among infants and children eligible for ART at enrolment was similar: overall, about three-quarters of infants and children started ART within six months. In the context of accelerated and universal ART access initiatives, it is important that children who engage in care be more efficiently initiated on ART, in order to prevent disease progression and death [19]. Encouragingly, the likelihood of ART initiation among infants and children in the most recent year (2012) was generally twice as high compared to 2010.

We found ART eligibility assessment was primarily done using WHO clinical staging. Fewer than one in four children received a CD4 cell count, a proportion significantly smaller than the >75% observed in other African settings [20]. While clinical assessment can be a valuable resource to identify severely ill children in need of immediate ART, it likely underestimates the proportion of children eligible to initiate treatment [21]. In our analysis, the proportion of ART eligible children increased by 6% when the few (<30%) children with CD4 cell count were included. Mainly basing on clinical assessment, 62% of under-five children were ART eligible at the time of enrolment. This proportion is comparable to the 63 to 88% reported in other African cohorts [20], but is likely to be an underestimate of all children eligible for ART at enrolment. Our analysis suggests that when the 2013 WHO guidelines are adopted (with universal ART initiation for under-five children) the proportion of children requiring ART would increase by 38%, and appropriate programme planning is needed to ensure all these children initiate ART and are retained on treatment.

About two-thirds of infants and ART-eligible children initiated ART within three months of enrolment. This proportion rose to three-quarters by six months and to 85% of infants and 81% of children at 12 months. This increase represents significant progress towards the WHO target, which during the study period was at least 80% of those who were ART eligible and receiving ART [22–24]. In light of more recent targets where >90% of the population should be on ART [5], additional efforts will be necessary to initiate more children on ART. Importantly, the gains in ART initiation may be offset by the significant lag between enrolment and ART initiation, even among infants where ART eligibility assessment was not a prerequisite to starting treatment. Notably, 38% of infants and close to half the children already had WHO Stage 3 and 4 disease at enrolment, highlighting the rapid progression of HIV disease in this age group [19, 25] and the urgency to initiate ART universally and promptly in the youngest age groups. Reasons for delayed ART initiation such as health system and patient/caregiver-related factors [26] will need to be addressed to ensure timely treatment for children. Of note, infants from PMTCT clinics were twice as likely to initiate ART, a finding that may suggest that the concerted efforts to increase early infant diagnosis and linkage to ART services [27–29] are beginning to bear fruit.

Infants and ART-eligible children enrolled most recently (in 2012) were generally twice as likely to initiate ART as those enrolled in 2010, suggesting eventual adoption of the 2009 guidelines. This success could be shadowed by a critical barrier to effective paediatric treatment: the delay between recommendations and change in practice, a well-described challenge in health systems that may already have inherent limitations [30]. This second lag combined with the delayed time to ART initiation of enrolled children discussed above could significantly compromise paediatric outcomes. Strategies for quicker adoption of guidelines and rapid field implementation of the recommendations are needed. We noted that primary health facilities performed well in initiating children on ART and private facilities had a higher likelihood of initiating infants on ART, highlighting the critical role of decentralization to lower levels and other facilities in the ART scale-up process [17]. We also observed that lacking a CD4 machine on site decreased the likelihood of ART initiation among children and was a potentially a barrier to ART initiation. It is expected that universal ART initiation for under-five children as recommended in the 2013 WHO guidelines will improve ART initiation among children, but our analysis suggests that it may take several years to observe the impact in the field.

The attrition on ART observed in our study (33% of infants and 28% of children) is similar to what has been observed in African countries [31–37]. However, other African cohorts suggest that lower rates of attrition are achievable among children [38–40]. Our study found severe malnutrition and advanced HIV disease correlated with ART attrition, a finding observed in other cohorts [9, 40, 41], highlighting the need to start ART before HIV disease progresses in children and mortality ensues. It also highlights the need to fully integrate and routinize nutrition assessment and nutrition support in paediatric ART programmes. Children enrolled from outpatient clinics had a higher risk of attrition, suggesting interventions may be necessary to strengthen linkages for these children. The challenge remains for Tanzania to implement interventions to further reduce attrition and promote timely initiation of ART among children.

This analysis has several strengths: it included a large cohort of recently enrolled children from multiple regions of Tanzania, and it reflects real-world implementation settings at a variety of health facilities. The analysis also combined patient and facility-level variables to assess association with key outcomes. Our study was limited in exploring reasons why children eligible for ART did not initiate ART, and this should be examined by further research of health systems and patient-level barriers to ART initiation. Using records of routinely collected data limits the ability to determine reasons for lack of important variables. For example, despite knowing that half the children attended clinics without a CD4 cell count machine, we could not definitively determine that CD4 cell count testing was not offered to the children, or blood taken and results not returned, or whether results were returned but not added to the medical records. Similarly, over the follow-up period, we found inadequate documentation of ART eligibility, but it is unclear whether this lack was due to poor documentation practices or limited eligibility assessment over the follow-up period. Follow-up clinical and laboratory assessments and completeness of data should be emphasized during ongoing supervision as paediatric ART services continue to be scaled up. Whereas the results may not be generalizable to other clinics that have not benefited from ICAP and PEPFAR support, the inclusion of patient and facility-level variables allowed us to control for variation of these characteristics in multiple settings.

## Conclusions

In this large cohort of under-five children, ART eligibility assessment using WHO clinical staging was a highly achieved first step in the paediatric ART cascade, but reliance on clinical staging likely underestimated children in need of ART. Findings suggest the majority of infants and children initiated ART in the first year. There was gradual adoption of the guidelines but important time lags observed: months between enrolment and ART initiation and years between recommendations and gradual change in practice, all of which can compromise paediatric outcomes. High attrition of infants remains a challenge in the context of universal ART recommendations, highlighting infant vulnerability to HIV infection and suggesting more efforts are needed to appropriately manage this age group.

## Supplementary Material

Implementation of antiretroviral therapy guidelines for under-five children in Tanzania: translating recommendations into practice
