# Supplementary material for: Implementation of antiretroviral therapy guidelines for under-five children in Tanzania: translating recommendations into practice
Source: J Int AIDS Soc. 2015 Dec 18;18(1):20303. doi: 10.7448/IAS.18.1.20303 (PMC4685962; doi:10.7448/IAS.18.1.20303)
Supplement: Implementation of antiretroviral therapy guidelines for under-five children in Tanzania: translating recommendations into practice [file JIAS-18-20303-s001.pdf]

Supplemental Tables: Implementation of antiretroviral therapy guidelines for children under 5 years in Tanzania

Table S1: Factors associated with being ART eligible at enrollment among HIV-infected children 12-59 months enrolled in HIV care between 2010 and 2012 in Tanzania (N=1210)

	univariate		multivariate	
	crude OR	95% CI	AOR	95% CI
**Age category**				
12-35 months	1.6	1.2-2.0	**1.5**	**1.2-2.0**
36-59 months	reference		reference	reference
**Sex**				
Male	reference		reference	
Female	1.1	0.8-1.3	1.1	0.9-1.4
**Enrolment year**				
2010	reference		reference	
2011	0.9	0.7-1.3	0.9	0.7-1.2
2012	1.3	0.9-1.7	1.2	0.9-1.7
**Point of entry**				
VCT	reference		reference	
PMTCT	1.2	0.9-1.7	1.2	0.9-1.6
Inpatient	2.0	1.4-2.8	**2.2**	**1.6-3.2**
Outpatient	1.1	0.7-1.6	1.2	0.8-1.9
Other/unknown	1.3	0.9-2.0	1.4	0.9-2.2
**Setting**				
Urban/semi-urban	1.9	1.2-2.9	**2.1**	**1.3-3.5**
Rural	reference		reference	
**Facility type**				
Primary	0.5	(0.4-0.8)	0.7	0.5-1.2
Secondary/tertiary	reference		reference	
Private/others	1.0	0.6-1.6	1.6	0.9-2.6
**CD4 machine onsite**	0.6	0.4-0.8	0.7	0.5-1.1

Note: Malnutrition, WHO stage and CD4 count excluded from model because they contribute to the definition of ART eligibility.

Table S2. Factors associated with pre-ART attrition among infants 0-11 months and children 12-59 months who enrolled at HIV clinics in Tanzania

	0-11 months (n=469)		12-59 months (n=1210)	
	Crude SHR(95% CI)	Adjusted SHR(95% CI)	Crude SHR(95% CI)	Adjusted SHR(95% CI)
**Age category**				
12-35 months			1.0 (0.8-1.3)	1.0 (0.8-1.3)
36-59 months			reference	reference
**Sex**				
Male	reference	reference	reference	reference
Female	0.9 (0.6-1.4)	1.0 (0.7-1.5)	1.2 (0.9-1.4)	1.2 (0.9-1.5)
**Malnutrition***				
Not Malnourished	reference	reference	reference	reference
Moderately malnourished	1.1 (0.6-2.1)	1.0 (0.6-1.9)	0.9 (0.5-1.4)	0.8 (0.5-1.3)
Severely malnourished	1.1 (0.7-1.8)	1.4 (0.7-2.5)	1.4 (1.0-2.0)	**1.4 (1.0-1.9)**
**Enrolment year**				
2010	reference	reference	reference	reference
2011	0.6 (0.4-1.0)	0.7 (0.4-1.2)	0.7 (0.6-1.0)	0.8 (0.6-1.1)
2012	0.3 (0.2-0.6)	**0.4 (0.2-0.8)**	0.4 (0.3-0.5)	**0.4 (0.3-0.6)**
**Missing enrolment CD4 cell count**	1.5 (0.6-4.1)	2.5 (0.8-8.1)	1.8 (1.3-2.3)	**2.0 (1.4-2.7)**
**WHO Stage**				
Stage I	reference	reference	reference	reference
Stage II	0.9 (0.5-1.4)	0.6 (0.4-1.1)	1.0 (0.7-1.4)	1.0 (0.7-1.4)
Stage III	1.0 (0.6-1.9)	08 (0.4-1.5)	0.7 (0.5-1.0)	0.7 (0.5-1.0)
Stage IV	1.3 (0.8-2.1)	0.6 (0.3-1.1)	1.5 (1.1-2.0)	1.1 (0.8-1.6)
**Point of entry**				
VCT	reference	reference	reference	reference
PMTCT	0.5 (0.3-0.9)	0.5 (0.3-0.9)	0.8 (0.4-1.5)	0.7 (0.4-1.2)
Inpatient	1.0 (0.5-1.8)	1.4 (0.8-2.4)	1.7 (1.2-2.4)	1.3 (0.8-2.2)
Outpatient	0.6 (0.4-1.1)	0.7 (0.4-1.2)	1.3 (1.0-1.8)	**1.6 (1.1-2.2)**
Other/unknown	0.5 (0.1-1.6)	0.5 (0.2-1.6)	0.8 (0.4-1.5)	0.9 (0.5-1.6)
**Location**				
Urban/semi-urban	1.4 (0.9-2.4)	1.1 (0.5-2.3)	1.6 (1.0-2.5)	1.6 (0.8-3.2)
Rural	reference	reference	reference	reference
**Facility type**				
primary	0.9 (0.5-1.4)	0.8 (0.5-1.5)	0.7 (0.5-1.0)	0.7 (0.4-1.2)
private and others	0.4 (0.4-0.7)	**0.4 (0.2-0.8)**	0.6 (0.4-1.0)	0.8 (0.5-1.2)
secondary/tertiary	reference	reference	reference	reference
**CD4 machine onsite**				
available onsite	reference	reference	reference	reference
available offsite	0.8 (0.5-1.4)	1.0 (0.5-1.8)	1.0 (0.6-1.6)	1.0 (0.6-1.6)
**Nutritional support**	1.3 (0.7-2.4)	1.3 (0.7-2.5)	0.9 (0.5-1.4)	0.9 (0.5-1.4)
**Outreach**	1.8 (1.1-3.0)	1.5 (0.9-2.3)	0.9 (0.5-1.6)	0.9 (0.5-1.6)

**Not Malnourished (Z score ≥-2); Moderately malnourished (Z score -2 to -3); Severely malnourished (Z score ≤ -3)*
